# Factors Influencing Quality of Life during the First Trimester of Pregnancy: A Prospective Cohort Study

**DOI:** 10.3390/medicina55100666

**Published:** 2019-10-01

**Authors:** Lina Jakubauskiene, Matas Jakubauskas, Antanas Mainelis, Diana Buzinskiene, Grazina Drasutiene, Diana Ramasauskaite, Tomas Poskus

**Affiliations:** 1 Faculty of Medicine, Vilnius University, 03101 Vilnius, Lithuania; morozovaite.lina@gmail.com (L.J.); matasjakub@gmail.com (M.J.); antanas.mainelis@santa.lt (A.M.); diana.buzinskiene@santa.lt (D.B.); grazina.drasutiene@santa.lt (G.D.); diana.ramasauskaite@santa.lt (D.R.); 2 Clinic of Obstetrics and Gynecology, Vilnius University Hospital “Santaros Klinikos”, 08410 Vilnius, Lithuania; 3 Center of Abdominal Surgery, Vilnius University Hospital “Santaros Klinikos”, 08410 Vilnius, Lithuania; 4 Faculty of Mathematics and Informatics, Vilnius University, 03225 Vilnius, Lithuania,

**Keywords:** quality of life, first trimester, pregnancy, prospective study, risk

## Abstract

*Introduction*: Pregnancy, delivery and postpartum periods are associated with fast changes leading to decreased self-confidence, anxiety, stress or even maternal depression impairing their quality of life (QOL). Although considered important, QOL of women during pregnancy is poorly understood. The aim of our study was to assess factors influencing QOL during first trimester of pregnancy. The secondary goal of our study was to evaluate whether QOL during first trimester of pregnancy is associated with newborn weight. *Materials and methods*: A prospective cohort study was performed including pregnant women during the first trimester visit. Our questionnaire consisted of the SF-36 QOL questionnaire, Wexner fecal incontinence scale, and other additional information. The SF-36 questionnaire mental (MCS) and physical (PCS) health scores were used in order to evaluate QOL of women during first trimester of pregnancy. Two multiple logistic regression models were created in order to determine independent variables that influence the QOL. *Results:* 440 pregnant women were included in the study. The two main domains that were used in the study were MCS and PCS, their medians were 50.0 (25.0; 50.0) and 50.1 (39.4; 59.0) points respectively. From the two logistic regression models we determined several independent factors that influence QOL of women during the first trimester of pregnancy. Additionally, we determined that women who reported worse QOL tended to give birth to newborns large for their gestational age. *Conclusions*: We found several significant variables that influence QOL of women during the first trimester of pregnancy. We also found that that lower MCS and PCS scores during the first trimester are associated with newborns large for gestational age.

## 1. Introduction

Pregnancy, delivery and postpartum periods are associated with fast hormonal, emotional and social changes, leading to decreased self-confidence, anxiety, stress or even maternal depression that can impact the health of both mothers and children [1,2,3].

Morbidity and mortality rates in obstetrics remain crucial for analyzing outcomes. However, improvement of maternal quality of life (QOL) during pregnancy is also very important from a population health standpoint. In recent years, QOL studies became a topic of interest in healthcare. QOL assessment is important for prevention and treatment programs [4]. Although considered important, QOL of women during pregnancy is poorly understood. Several studies investigating QOL of pregnant women focus only on specific illnesses, such as gestational diabetes mellitus, hypertension, and depression, and overlook daily socioeconomic factors that influence the QOL in the general population of pregnant women [5]. A recent review by Lagadec et al. shows that maternal QOL tend to decrease or remain stable throughout pregnancy [6]. With this in mind and the fact, that interventions to improve quality of life take time to provide results, interventions must start as early as possible—before conception or in the early periods of pregnancy [7,8]. The aim of our study was to assess factors influencing QOL during the first trimester of pregnancy. The secondary goal of our study was to evaluate whether QOL during the first trimester of pregnancy is associated with newborn weight.

## 2. Materials and Methods

We performed a prospective cohort study, which was approved by the Vilnius Regional Biomedical Research Ethics Committee (date of approval 2009-07-10, reference number: 158200-7-059-13). The study design is presented in detail in the publication by Poskus et al. [9].

### 2.1. Study Population

In this study we included pregnant women of 18–45 years of age, who gave birth in Vilnius University Hospital “Santaros Klinikos” between January 2010 and December 2011 and signed the consent form to participate. Women enrolled in this study were scheduled for a total of four visits (first trimester, third trimester, 1–2 days after delivery and a month after delivery). During all four visits the same gynaecologist (DB) interviewed and examined the women. Our period of interest for this publication is the primary visit during the first trimester of pregnancy when the participating women filled out a detailed questionnaire.

### 2.2. Questionnaire

Our questionnaire consisted of a SF-36 QOL questionnaire, Wexner fecal incontinence scale and other additional information, that can be classified into these groups: demographics, anthropometric data, dietary habits, use of oral supplements, use of alcohol and tobacco, exposure to tobacco fumes, physical activity, working conditions, living environment, obstetric history, perianal symptoms, defecation history and the presence of chronic health conditions.

### 2.3. Statistical Analysis

We used the SF-36 questionnaires mental (MCS) and physical (PCS) health scores to evaluate the women’s QOL during the first trimester of pregnancy. In order to determine independent variables that influence the QOL we created two multiple logistic regression models (one for MCS and the other for PCS scores) using the stepwise method analyzing every possible variable from the questionnaire. A *p*-value of <0.05 was set for entry and removal of the variable from the model. Furthermore, using Kruskal–Wallis and Dunn’s post hoc tests, we looked into the relationship between QOL and newborn weight groups (low birth weight <10 percentile; normal weight 10–90 percentile and large for gestational age >90 percentile) according to gestational age and sex (in this analysis we did not include women who were lost on follow-up). Statistical analyses were performed using SPSS^®^ software version 21 (IBM, Armonk, NY, USA).

## 3. Results

A total of 440 pregnant women were included in the study. The mean age was 28.7 ± 5.6 years. Out of 440 women, 208 (47.3%) were primiparous and 232 (52.7%) were multiparous. Demographic and socio-economic results are presented in Table 1.

Median values of all SF-36 questionnaire domains are presented in Table 2. The main two domains that further are used in the study are MCS and PCS, with their medians respectively 50.1 (IQR 39.4–59.0) and 48.2 (IQR 41.0–63.3) points.

Table 3 represents the PCS multiple logistic regression model. ANOVA p-value for the whole model is <0.001 and R^2^ = 0.804.

MCS multiple logistic regression model is presented in Table 4. ANOVA p value for the whole model is <0.001 and R^2^ = 0.779.

Additionally, we analyzed whether women’s QOL during the first trimester of pregnancy is related to the weight of the newborn (Table 5). We did not include 160 women (36.4%), who were lost to follow-up. We found that women who evaluated their QOL to be lower tended to give birth to newborns who were large for gestational age.

## 4. Discussion

We analyzed maternal QOL during the first trimester of pregnancy in this study. Women in our study scored similarly in SF-36 subscale scores compared to the two other related studies [10,11]. We found several factors influencing reported QOL and we present them in two logistic regression models, one explaining the influence of factors on PCS and the other on MCS.

We determined that, among dietary factors, coffee consumption had a negative influence both for PCS and MCS. This could be partly explained by the fact that caffeine found in coffee acts as a stimulant and can cause symptoms similar to anxiety disorders (irritability, nervousness) [12]. Alcohol consumption tended to improve MCS of pregnant women. Lacasse et al. report identical results [13]. There is no clear interpretation for this finding, however, some authors suggest that moderate amounts of alcohol reduce stress [14,15]. Although alcohol use may look beneficial, it has a lot of negative effects on pregnancy outcome, and moreover it can lead to fetal alcohol spectrum disorder [16,17]. Therefore, we would strongly advice against the use of alcohol during pregnancy. We also found several dietary factors that negatively affect MCS or PCS and are hardly explained by the current evidence in literature. For example, fruit and vegetable consumption reduces both PCS and MCS, and warm food and fish intake reduces PCS alone. We want to address our findings regarding oral supplements separately as they are widely used in pregnancy. We found that only iron supplements have positive impact on MCS, whereas the use of folic acid significantly reduces MCS. In addition to this, our findings suggest that the use of all other oral supplements have only negative effects on PCS. It is proven that iron supplements improve the well-being of women during pregnancy by preventing iron deficiency anemia, which is very prevalent in pregnant women [18].

We thought that all perianal symptoms would be associated with worse QOL, however, to our surprise we found several perianal variables that significantly increase PCS and MCS. These include straining during defecation (PCS and MCS) and fecal incontinence that requires change in lifestyle (MCS only). Johannessen et al.’s study completely contradicts this finding, as they determined that fecal incontinence during pregnancy greatly impairs quality of life in the psychological domain [19].

The number of pregnancies significantly reduces PCS; this has been reported in many times in the literature. A study by Hama et al. reports that multiparous women report better QOL during pregnancy and a study by Chang et al. report opposite results regarding parity [11,20]. Weight gain during pregnancy negatively affects only MCS. One study refers that weight gain in pregnancy can cause discomfort in general [21]. History of perineal tear during delivery has a negative impact on both MCS and PCS, this was also proven in a study by Monard et al. [22]. Perineal tear in some cases can be partly avoided by choosing episiotomy or by performing antenatal perineal massage [23,24]. However, the latter method is debatable [25].

Cold working environment was associated with higher QOL (both MCS and PCS). We can only speculate on the causes, but it is known that during pregnancy women experience hormonal changes, especially fluctuation of estrogen levels, which can lead to hot flushes, and thus lower temperatures could be more comfortable [26]. We also analyzed how physical activity affects QOL. Weekly exercises tend to increase MCS of pregnant women, however vigorous physical activity and lifting more than 10 kilograms daily reduces PCS and MCS respectively. Guidelines on physical activity during pregnancy differ throughout countries, some of them allow intensive physical activities [27], however, most of them recommend moderate daily exercises [27,28]. Intensive physical activity before pregnancy can become unbearable throughout pregnancy due to decline in physical functioning [29,30,31].

We also analyzed whether QOL during the first trimester of pregnancy is associated with newborn weight. We found that women with lower PCS and MCS scores tended to give birth to newborns who were large for their gestational age. This could be clinically relevant, as large for gestational age newborns and their mothers tend to have more complications during labor [32,33,34]. Current evidence in the literature is limited, but suggests that worse QOL is associated with higher oxidative stress and lower birthweight [35].

The strengths of this study are the prospective nature of our collected data and the application of a standardized QOL questionnaire SF-36. The main advantage is the analysis of a very broad spectrum of variables that can impact women’s QOL, this allows us to accurately produce a predictive regression model.

One of the drawbacks of our study is the narrow period of interest (only the first trimester of pregnancy). As the pregnancy grows, women face more physiological changes, therefore analysis of the other two trimesters could be as, if not more, important in identifying other variables influencing women’s QOL.

Findings from our study could be important in clinical practice as some of the variables determined can be modified. Different interventions can be developed to improve QOL of pregnant women. Interventions to improve quality of life take time to bring results, therefore actions must start as early as possible— before conception or in the early pregnancy periods [8]. The literature suggests such interventions are most effective in the earlier, periconceptional, period, as this is a key time for fetal development [7].

## 5. Conclusions

In conclusion, we found several significant variables that impact women’s QOL during the first trimester of pregnancy. Some of the variables are new and their interactions can be difficult to explain, thus further investigation is needed to explore them. The modifiable risk factors we determined could be important in clinical practice for developing and promoting interventions to improve maternal QOL in the first trimester, as this is a key time for fetal development. Moreover, we acknowledge that lower QOL during first trimester is associated with newborns who are large for gestational age.

## Figures and Tables

**Table 1:** General demographic and clinical data.

Characteristics	Study Group (*n* = 440)
Patients’ age (years) (mean ± SD)	28.7 ± 5.6
BMI before pregnancy (kg/m^2^) (median (range))	23.1 (15.4–43.8)
Gravidity (*n* (%)) Nulligravida Multigravida	199 (45.2%) 241 (54.8%)
Parity (*n* (%)) Nulliparous Multiparous	208 (47.3%) 232 (52.7%)
Marital status (*n* (%)) Single Married or partnership	107 (24.3%) 333 (75.7%)
Maternal education (*n* (%)) High school Incomplete university or college degree University of college degree	60 (13.6%) 167 (38.0%) 213 (48.4%)
Living area (*n* (%)) Urban Rural	351 (79.8%) 89 (20.2%)
Monthly household income (*n* (%)) Less than average (<850 euros) Average (850 euros) Above average (more than 850 euros)	52 (11.8%) 326 (74.1%) 62 (14.1%)

SD, standard deviation.

**Table 2:** SF-36 questionnaire general results.

SF-36 Domains	Study Group (*n* = 440)
Physical function (median (IQR))	75.0 (60.0–100.0)
Physical role function (median (IQR))	50.0 (0.0–100.0)
Emotional role function (median (IQR))	66.7 (0.0–100.0)
Vitality (median (IQR))	50.0 (40.0–65.0)
Emotional well-being (median (IQR))	52.0 (40.0–68.0)
Social functioning (median (IQR))	50.0 (37.5–100.0)
Bodily pain (median (IQR))	55.0 (45.0–100.0)
General health perceptions (median (IQR))	45.0 (40.0–50.0)
Perceived change in health (median (IQR))	50.0 (25.0–50.0)
Mental health score (MCS) (median (IQR))	50.1 (39.4–59.0)
Physical health score (PCS) (median (IQR))	48.2 (41.0–63.3)

IQR, Interquartile range.

**Table 3:** Multiple logistic regression model for factors influencing physical (PCS) health scores.

Variable	Unstandardized Beta Coefficients	*p-*Value
(Constant)	78.404	<0.001
**Dietary**		
Coffee consumption	−2.539	0.002
How often fish and fish products are consumed weekly	−1.430	0.001
How often meat and meat products are consumed weekly	0.968	0.020
How often fruits and vegetables are consumed weekly	−0.883	0.011
How often milk and dairy products are consumed weekly	0.922	0.025
How often warm food is eaten during the day	−1.556	<0.001
Taste is the main criteria when choosing groceries	−2.179	<0.001
Snacking between meals	−4.994	0.001
Use of oral nutritional supplements (except folic acid and iron supplements)	−2.662	<0.001
**Peri-anal symptoms**		
Perianal pain during pregnancy	−7.600	<0.001
Defecation less than 3 times a week	−6.590	<0.001
Straining during defecation	3.509	<0.001
Pain during and after defecation	−2.732	0.002
**Physical variables**		
Current body weight	−0.060	0.016
Rh(D) positive blood group	−2.106	0.006
**Obstetric history**		
History of perineal tear during delivery	−3.768	<0.001
Number of pregnancies	−1.978	0.001
**Socioeconomic variables**		
Cold working environment	4.317	<0.001
Good living conditions	3.147	<0.001
Living in an urban area	−2.563	0.002
Secondhand smoking	1.848	0.005
Noisy environment more than 6 hours a day	−3.305	0.010
Lifting more than 10 kilograms daily	−4.374	0.001

**Table 4:** Multiple logistic regression model of factors influencing mental (MCS) health scores.

Variable	Unstandardized Beta Coefficients	*p-*Value
(Constant)	57.079	<0.001
**Dietary**		
Coffee consumption	−1.861	0.031
Alcohol consumption	2.653	0.001
How often fruits and vegetables are consumed weekly	−1.905	<0.001
How often grain products are consumed weekly	−0.938	0.010
How often products containing flour are consumed weekly	1.370	0.001
Meals eaten daily	0.908	0.038
Use of iron supplements	1.632	0.010
Use of folic acid supplements	−2.006	0.016
**Peri-anal symptoms**		
Perianal pain during pregnancy	−5.464	<0.001
Defecation less than 3 times a week	−4.744	<0.001
Straining during defecation	3.574	<0.001
Obstacle feeling during defecation	−3.239	0.001
Fecal incontinence that requires a pad	−6.281	<0.001
Fecal incontinence that requires to change the lifestyle	6.129	0.002
**Obstetric history**		
History of perineal tear during delivery	−2.035	0.004
Body weight gain during pregnancy	−0.108	0.044
**Socioeconomic variables**		
Exercising weekly	1.364	<0.001
Too intensive physical activity	−8.427	0.019
Emotional distress at work	−1.868	0.002
Working with computer	2.627	<0.001
Dusty working environment	−2.952	0.005
Cold working environment	2.603	0.026

**Table 5:** Relationship between first trimester QOL and newborn weight.

QOL Variables	Newborn Weight			*p-*Value		
	Low Birth Weight (<10 Percentile) (*n* = 17)	Normal Weight (10–90 Percentile) (*n* = 226)	Large for Gestational Age (>90 Percentile) (*n* = 37)	Low Birth Weight vs. Normal Weight	Low Birth Weight vs. Large for Gestational Age	Normal Weight vs. Large for Gestational Age
MCS (median (IQR))	48.2 (40.1–55.5)	42.1 (39.5–53.44)	39.4 (39.0–40.4)	0.599	<0.001	<0.001
PCS (median (IQR))	51.44 (40.8–63.5)	43.0 (40.0–63.4)	41.0 (39.3–41.4)	0.413	0.001	<0.001
